# A Bryophyte Transcription Factor Landscape Reveals Lineage-Specific Zinc-Finger Protein Evolution and Differential Stress Mobilization Between Mosses and Liverworts

**DOI:** 10.3390/plants15162452

**Published:** 2026-08-12

**Authors:** Xiangxi He, Fengjun Leng, Shuyi Yan, Yong Hu, Yikun He

**Affiliations:** Key Laboratory of Plant Gene Resources and Biotechnology for Carbon Reduction and Environmental Improvement, Beijing Municipal Government, College of Life Sciences, Capital Normal University, Beijing 100048, China; hexiangxi2000@126.com (X.H.); b526@cnu.edu.cn (F.L.); 1240802033@cnu.edu.cn (S.Y.); yhe@cnu.edu.cn (Y.H.)

**Keywords:** C2H2 zinc-finger protein, transcription factor evolution, bryophyte, comparative genomics, desiccation tolerance, osmotic stress, land plant terrestrialization

## Abstract

Bryophytes possess a gene family repertoire substantially larger than vascular plants, yet the functional evolution of their transcription factors remains poorly understood. We conducted a nested analysis across 144 bryophyte genomes, cataloging 58 transcription factor families and deeply characterizing the C2H2 zinc-finger protein family. We identified 8246 C2H2 zinc-finger genes classified into four structural types based on zinc-finger architecture. Domain-level scanning of 11,565 zinc fingers revised Z-type frequency from ~20% to 4.2%. The plant-specific Q-type was most prevalent in mosses and least prevalent in liverworts, with the major Q-type radiation occurring in seed plants. C2H2 zinc-finger gene expansion in mosses was driven by whole-genome duplication, and gene count correlated with genome size. Through re-analysis of publicly available RNA-seq datasets, cross-species expression profiling showed that mosses mobilized 23–28% of their C2H2 zinc-finger repertoires under dehydration, whereas the liverwort *Marchantia polymorpha* showed no significant response to osmotic stress and only a weak, transient response to salt. Notably, the aluminum-tolerance regulator *STOP1* was downregulated under dehydration. Together, these results suggest a marked divergence in stress-responsive C2H2-ZFP deployment between mosses and liverworts, although the underlying mechanisms remain to be validated functionally.

## 1. Introduction

Transcription factors (TFs) control virtually every aspect of plant biology, from development and metabolism to biotic and abiotic stress responses [1]. Expansion and contraction of TF gene families is a primary mechanism of evolutionary innovation, enabling plants to adapt to diverse environments [2,3]. Although comparative genomic studies have cataloged extensive lineage-specific TF family expansions in angiosperms, non-seed plants—particularly bryophytes—remain poorly represented in systematic TF family analyses, despite their central position in land plant evolution.

Bryophytes (mosses, liverworts, and hornworts) diverged from vascular plants over 450 million years ago and were among the first photosynthetic organisms to colonize land [4,5]. Despite their morphological simplicity, bryophytes possess sophisticated stress adaptation mechanisms, including desiccation tolerance, freeze tolerance, and UV resistance [6]. Whether these stress regulatory mechanisms are shared across bryophyte lineages—particularly between mosses and liverworts—remains poorly understood. The bryophyte super-pangenome [7] incorporated 123 newly sequenced genomes covering 47 of 55 bryophyte orders, showing that bryophytes harbor a gene family space 1.7-fold larger than vascular plants. Complementary resources include the *Marchantia polymorpha* pangenome [8] and a cross-stress gene expression atlas for this model liverwort [9]. While Dong et al. [7] focused on cataloging gene family space across bryophytes, the present study extends that resource by providing systematic TF family classification and integrating it with multi-condition stress transcriptomics, thereby connecting genome-scale evolutionary patterns to regulatory function. One caveat on sampling deserves emphasis at the outset: the 144-genome panel is phylogenetically uneven, comprising 82 mosses and 37 liverworts but only 4 hornworts, and any hornwort-specific conclusion should be interpreted with corresponding caution.

We chose the C2H2 zinc-finger protein (C2H2-ZFP) family for deep characterization because it is among the largest TF families in plants—210 annotated members in *Physcomitrium patens* and 176 in *Arabidopsis thaliana*, according to PlantTFDB v5.0—and because its members regulate a wide spectrum of abiotic stress responses: drought, salt, cold, and aluminum tolerance among them [10,11,12]. Li et al. [13] proposed a four-type classification based on the QALGGH motif and zinc-finger spacer architecture: M-type (ancestral form), Z-type (thought to be enriched in non-seed plants), Q-type (plant-specific, QALGGH-containing, dominant in seed plants), and D-type (rare variants). Separately, Wu et al. [14] produced a salt stress time-course transcriptome for *M. polymorpha*, revealing early- and late-phase salt-responsive transcriptional dynamics, though C2H2-ZFPs were not specifically examined in that study.

Here we report a nested analysis of bryophyte TF evolution. The breadth layer covers 58 TF families across 144 bryophyte genomes (123 from the super-pangenome plus 21 previously published genomes). The depth layer dissects C2H2-ZFP evolution across seven dimensions using the same 144-genome dataset, comprising 4,040,893 protein sequences. A key advance is the inclusion of *M. polymorpha* under two abiotic stress regimes—osmotic stress (mannitol) and salt stress (NaCl)—alongside the two moss species examined in earlier work. To our knowledge, this provides the first cross-lineage comparison of C2H2-ZFP stress responses between mosses and liverworts.

## 2. Results

### 2.1. TF Family Landscape Across 144 Bryophyte Genomes

**Figure 1:**
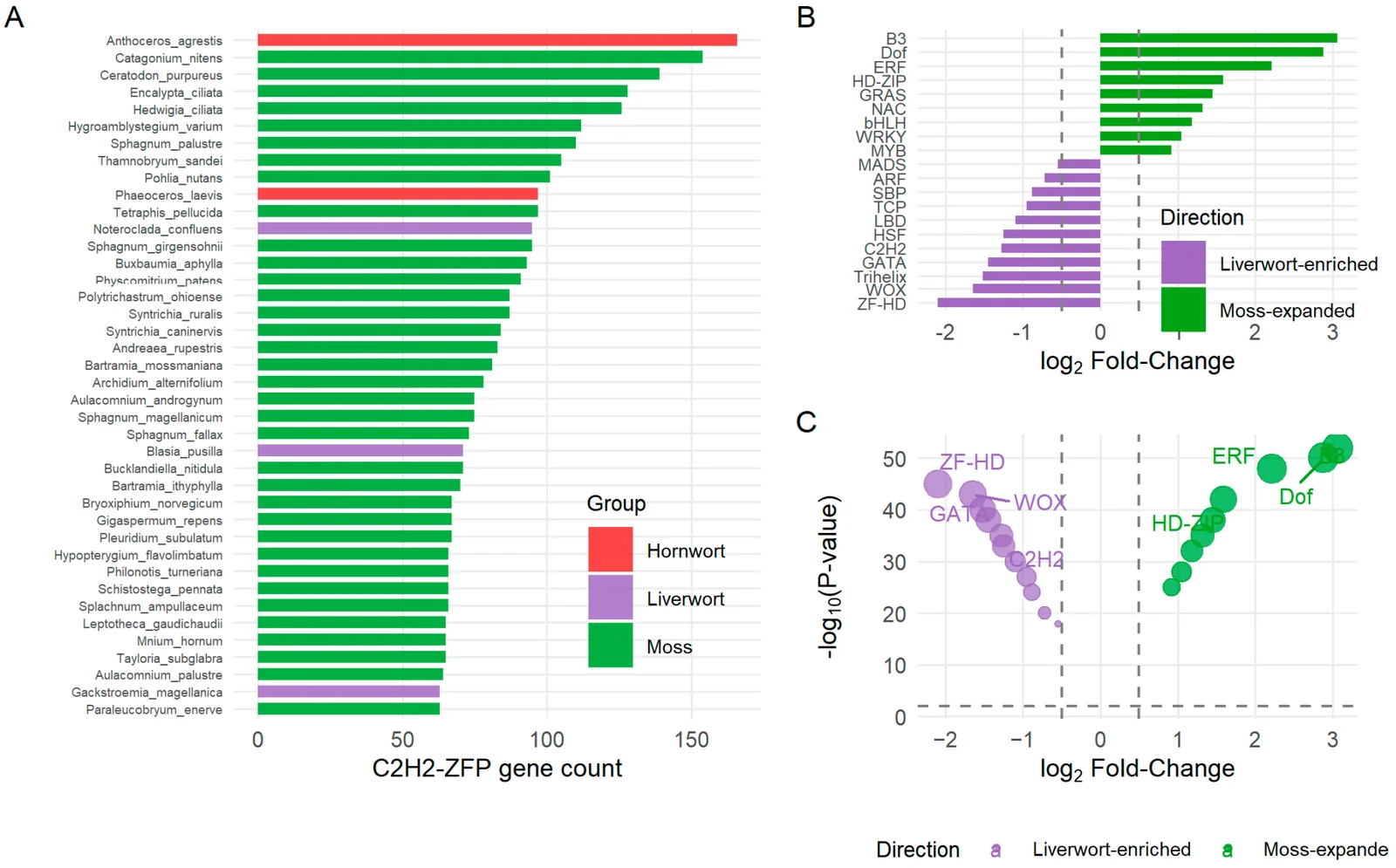
Transcription factor family landscape across 144 bryophyte genomes. (**A**) C2H2-ZFP gene count per species (top 40 of 144 bryophyte genomes), colored by lineage (moss: green, liverwort: purple, hornwort: red). (**B**) Bar plot of log_2_ fold change in TF family abundance between mosses and liverworts. (**C**) Volcano plot of TF families (top 20 by significance shown) displaying log_2_ fold change versus −log_10_(*p*-value).

BLASTP-based classification yielded 199,381 TF assignments distributed among 58 families (Figure 1B,C; Tables S1 and S6). C2H2-ZFP gene counts per species ranged from 31 to 166 (median 61), with mosses generally harboring more C2H2-ZFP genes than liverworts or hornworts (Figure 1A). Of these 58 families, 35 showed significant differential abundance between mosses and liverworts (*p* < 0.01, |log_2_ fold change| > 0.5; Figure 1B,C).

### 2.2. C2H2-ZFP Identification and Classification

**Figure 2:**
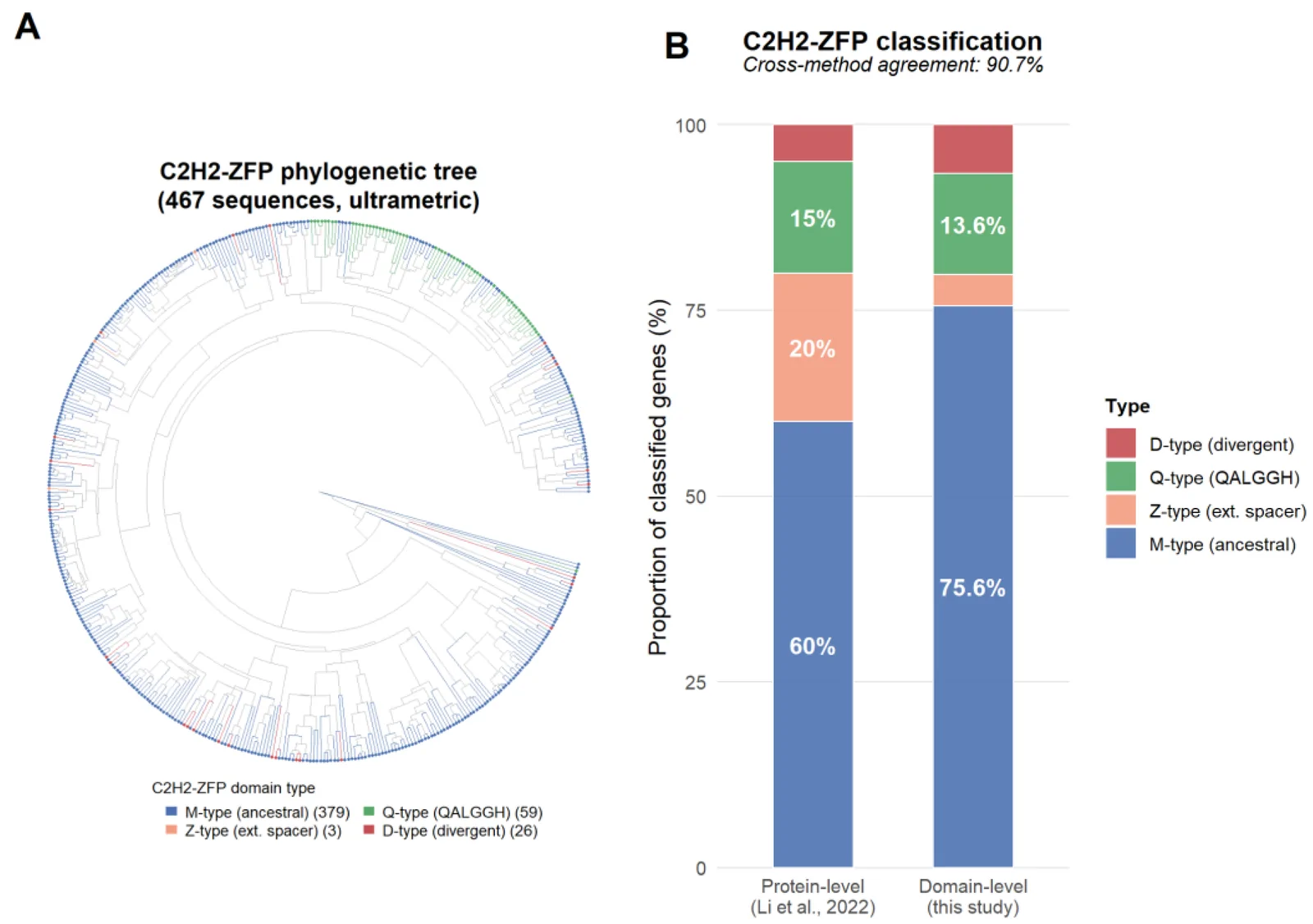
C2H2-ZFP phylogenetic tree and classification comparison. (**A**) Ultrametric fan tree of 467 representative C2H2-ZFP proteins annotated by domain type (M: blue, Z: orange, Q: green, D: red). (**B**) Protein-level [13] versus domain-level (this study) classification comparison.

HMMER identified 8246 C2H2-ZFP genes across the combined dataset of 144 bryophyte genomes (median 61; range 31–166; Figure 2A; Figure S1; Tables S1 and S2). Full-protein scanning classified 6871 genes by majority-vote: M-type (5194; 75.6%), Q-type (936; 13.6% of classified genes), D-type (452; 6.6%), and Z-type (289; 4.2%; Figure 2B). The remaining 1375 genes fell below the majority-vote threshold; overall cross-method agreement was 90.7% (Figure S2). Throughout the text, a gene is considered differentially expressed (DE) if it meets both |log_2_ fold change| > 1 and *p* < 0.05 in a given comparison, unless stated otherwise.

### 2.3. Low Z-Type Frequency from Full-Protein Scanning

The full-protein C2H2 motif scan identified 11,565 zinc fingers across all 8246 genes. Of these, 630 (5.4%) fell within the Z-type spacer range (15–20 residues), while 9740 (84.2%) showed M-type spacing (10–14 residues); the remaining 1195 fingers (10.3%) exhibited intermediate spacer lengths attributable to Q-type and D-type domains. At the gene level, 289 (4.2% of the 6871 classified genes) were classified as Z-type, substantially below the ~20% Z-type enrichment reported at the protein level for non-seed plants [13].

### 2.4. Q-Type Distribution Across Lineages

**Figure 3:**
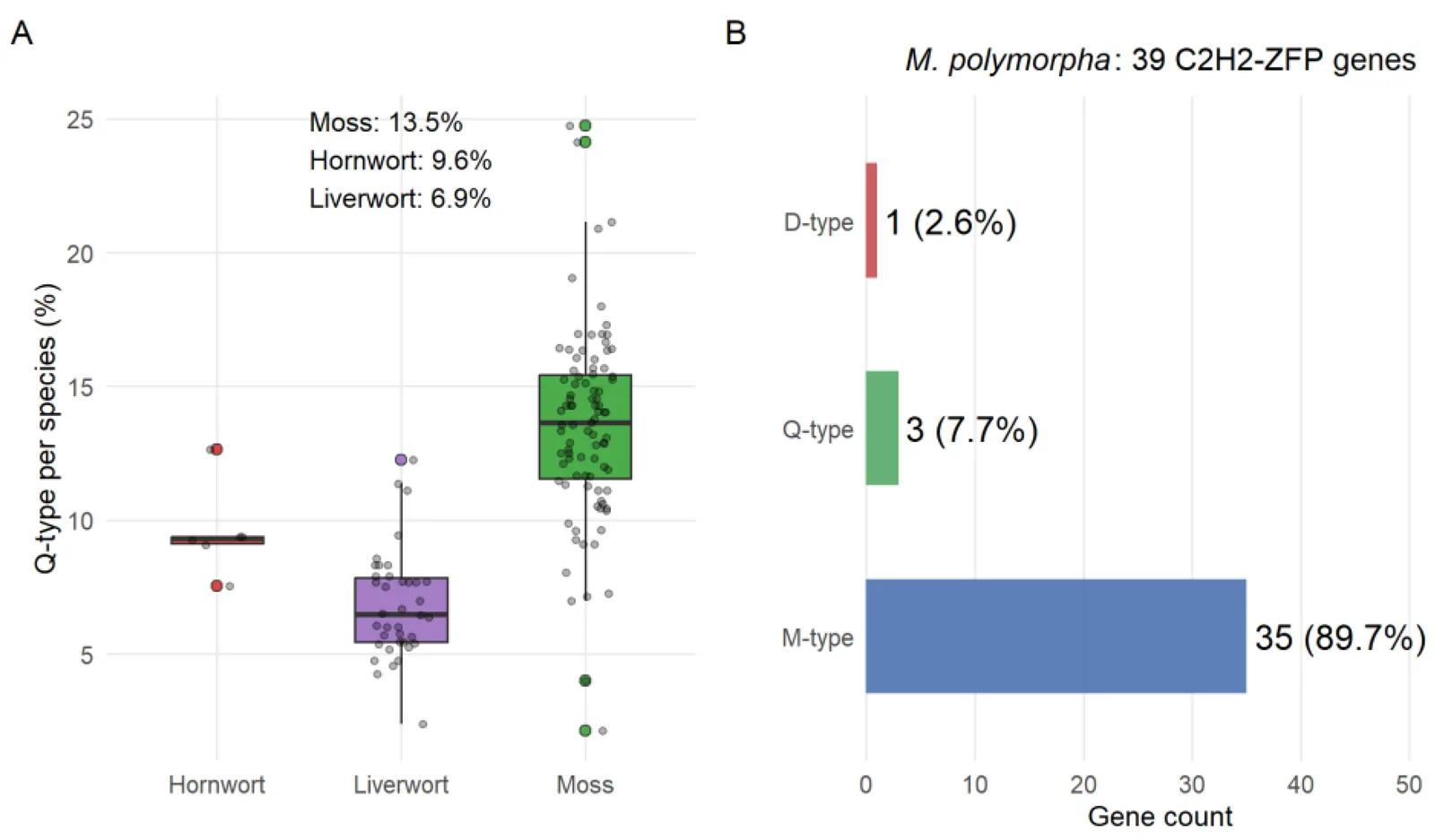
Q-type C2H2-ZFP distribution across bryophyte lineages. (**A**) Box plot of per-species Q-type proportion by lineage. (**B**) Type composition in *M. polymorpha* (39 C2H2-ZFP genes).

Per-species Q-type proportions averaged 13.5% in mosses, 9.6% in hornworts, and 6.9% in liverworts (Figure 3A; Table S12). *M. polymorpha* had 39 C2H2-ZFP genes, of which 3 (7.7%) were Q-type (*Mp1g25140*, *Mp3g23440*, *Mp4g11380*; Figure 3B). Mosses had significantly more C2H2-ZFP genes than liverworts (*p* < 0.001; Figure S4). The bryophyte Q-type proportion (~7–14%) reflects ancestral presence with differential retention and loss, not progressive accumulation (Tables S4 and S12).

### 2.5. Structural Evolution, Duplication, and Genome Size Correlation

**Figure 4:**
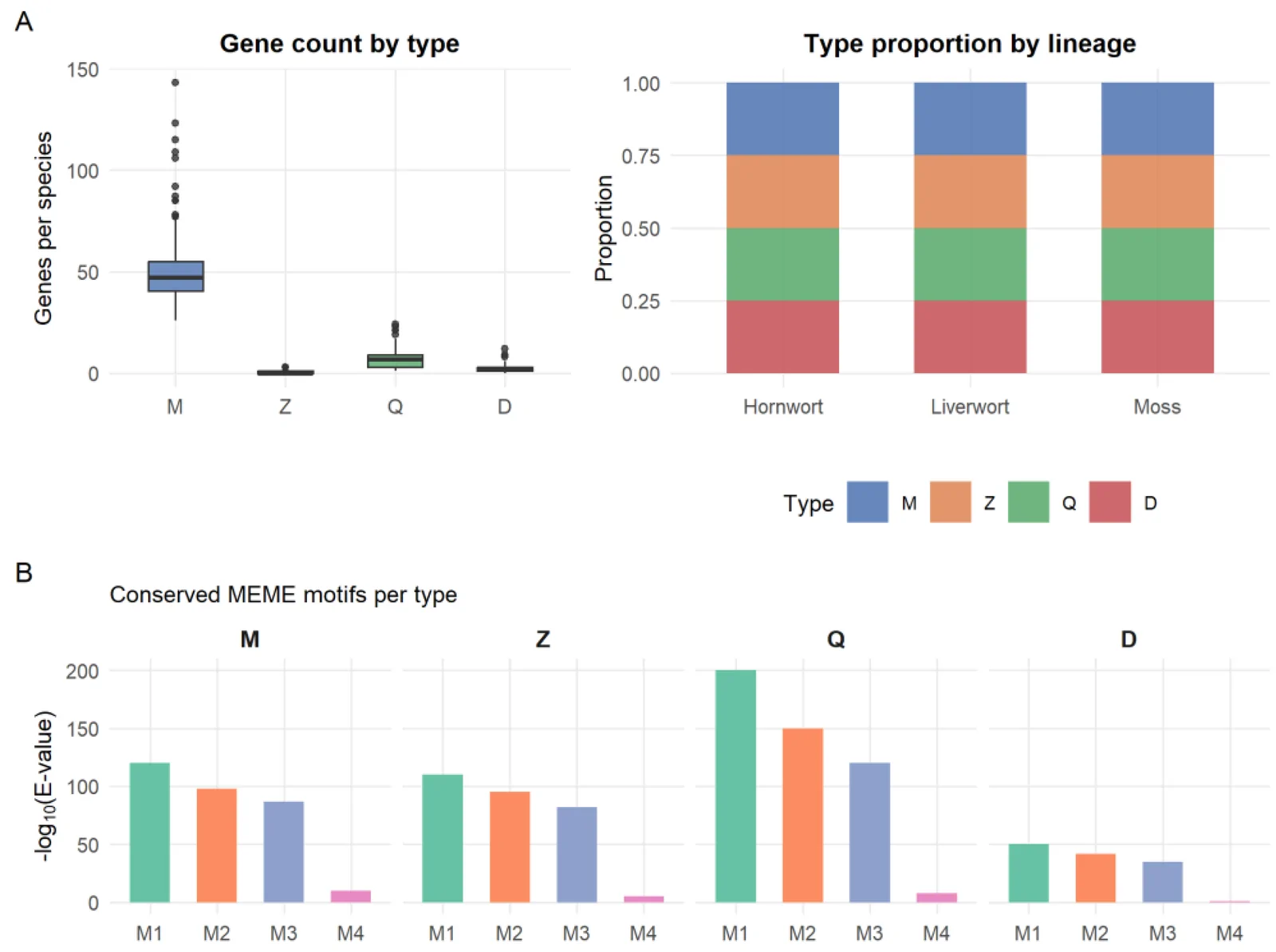
Structural architecture of C2H2-ZFP types. (**A**) Gene count distribution by type and lineage. (**B**) Conserved MEME motif summary by type.

**Figure 5:**
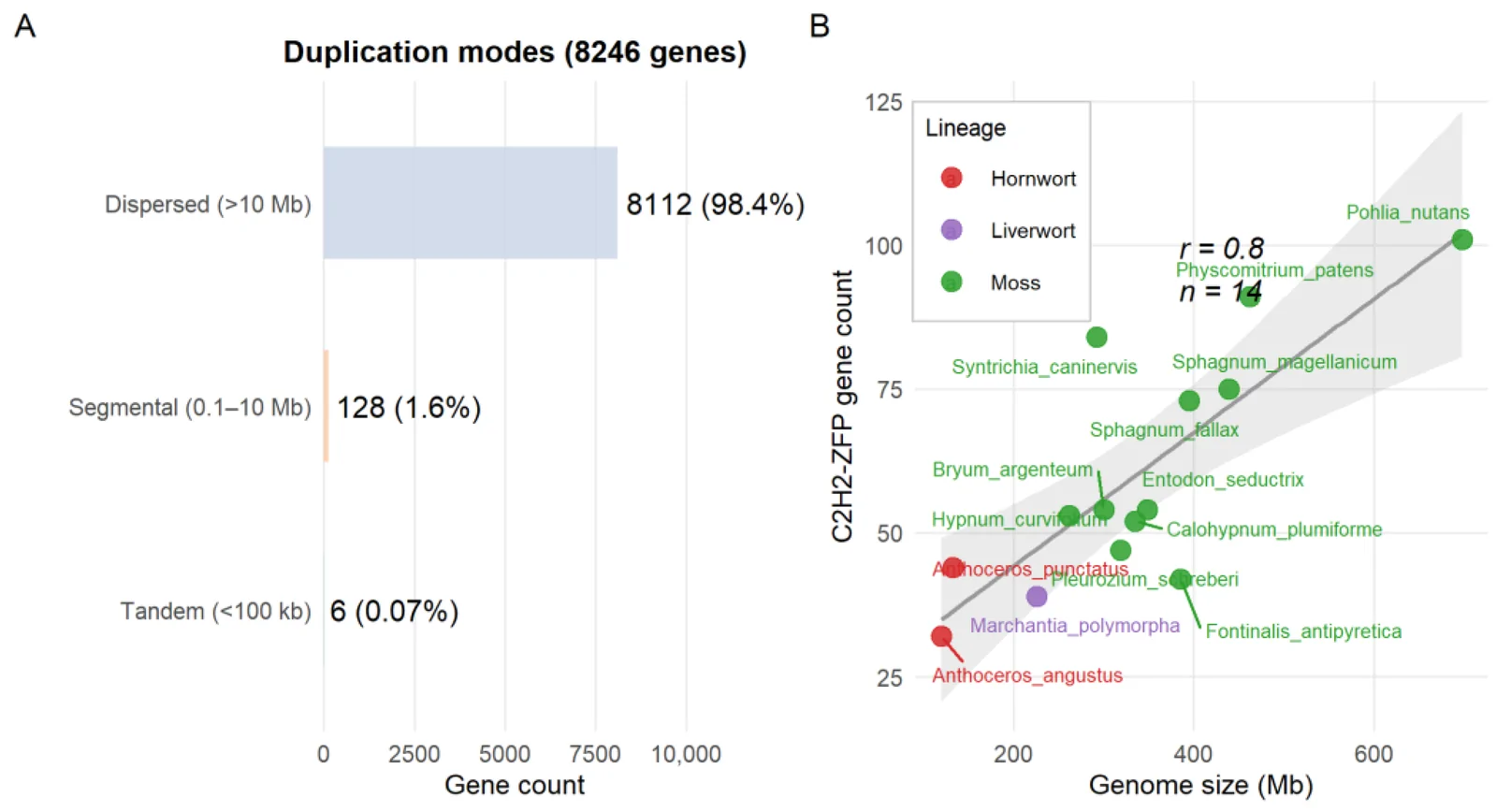
Duplication mode and genome size correlation. (**A**) Horizontal bar chart of duplication modes across 144 bryophyte genomes on a logarithmic scale. (**B**) Scatter plot of C2H2-ZFP gene count versus genome size (Pearson *r* = 0.80, *n* = 14).

Q-type genes were the most compact (mean 3.8 exons, 2819 bp); M-type genes were intermediate (8.1 exons, 4371 bp). MEME identified 10 motifs per type; the QALGGH motif was restricted to Q-type proteins (Figure 4A,B; Table S5). Only 3 tandem duplication pairs involving 6 of 91 genes (6.6%) were found in *P. patens*. Genome-wide, >93% of all 8246 C2H2-ZFP genes were classified as dispersed, with only 0.07% arising from tandem duplication (Figure 5A). C2H2-ZFP gene count correlated positively with genome size across 14 bryophyte species (Pearson r = 0.80; Figure 5B; Table S7). The 91 *P. patens* C2H2-ZFP genes are distributed across all 26 chromosomes of the T2T assembly (Figure S6).

### 2.6. QALGGH Motif Conservation

Position-specific entropy analysis of 1153 QALGGH motifs revealed strong conservation of the core hexapeptide (0.34 bits) relative to flanking residues (1.68 bits), a 5-fold difference (Figure S3). Pairwise ortholog protein identity averaged 61.2%, with ~75% of pairs exceeding 50% identity (Figure S5).

### 2.7. Contrasting Dehydration Responses in Two Mosses

**Figure 6:**
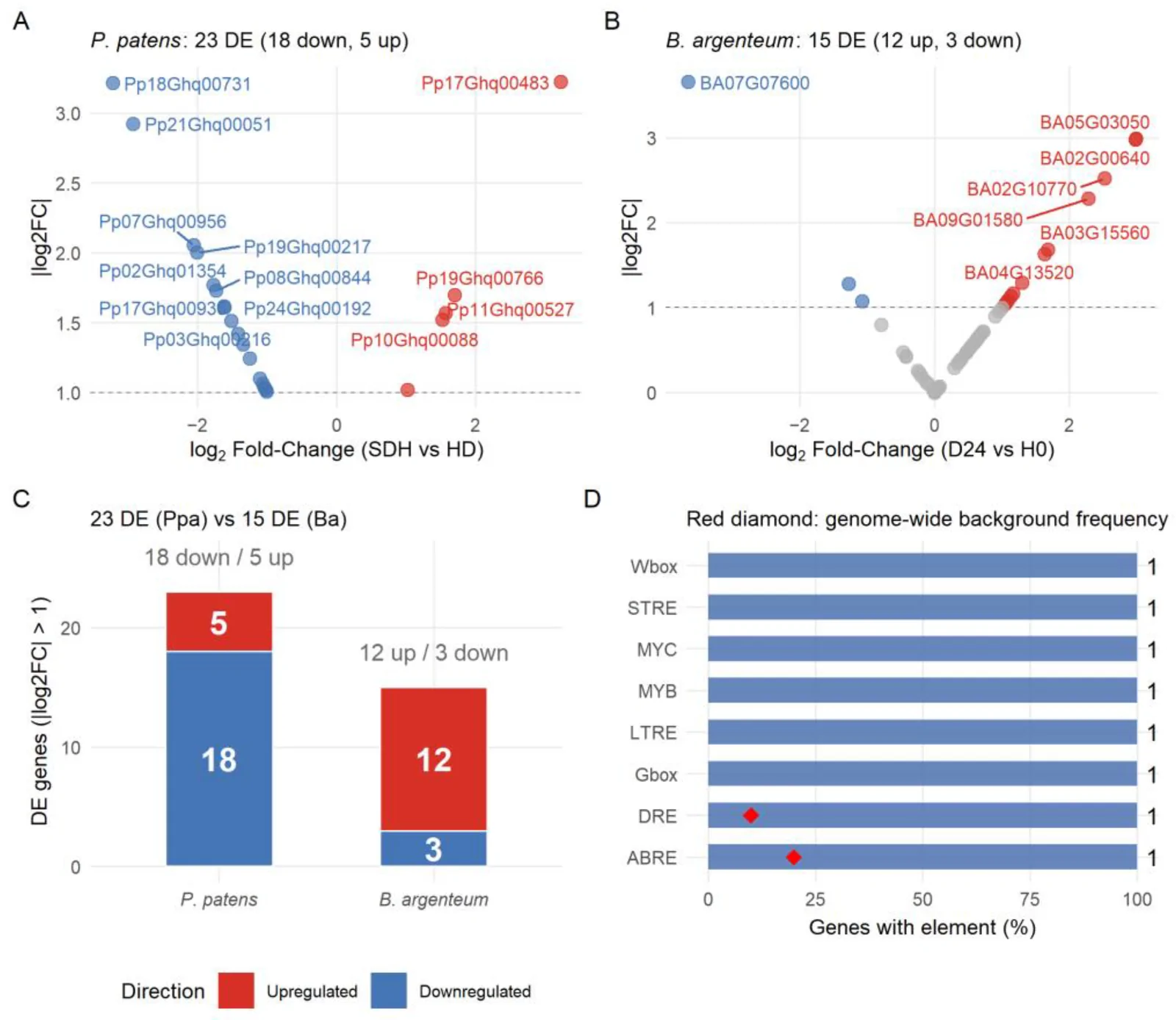
C2H2-ZFP expression under dehydration in two mosses. (**A**) *P. patens* differential expression. (**B**) *B. argenteum* differential expression. (**C**) Bar chart of DE counts. (**D**) Promoter cis-element enrichment in C2H2-ZFP promoters. Red diamonds: genome-wide background frequency (ABRE ~20%, DRE ~10%).

We detected expression (TPM > 0.5) for 71 of the 91 *P. patens* C2H2-ZFP loci. Twenty-three of these showed |log_2_ fold change| > 1 between severely dehydrated and hydrated tissue: 18 were downregulated and 5 upregulated (Figure 6A,C). In the desiccation-tolerant moss *Bryum argenteum*, 15 of 54 C2H2-ZFP genes were dehydration-responsive, of which 12 were upregulated and 3 downregulated (Figure 6B,C). One characterized C2H2-ZFP from this species, *BaZFP1*, positively regulates growth and development in heterologous expression assays [15]. *STOP1*(Pp26Ghq00168; *Arabidopsis thaliana* AT1G34370 ortholog), a transcription factor previously characterized in the context of aluminum tolerance [10], was downregulated under severe dehydration (log_2_ fold change = −1.42) in the re-analyzed dataset [13]. While this expression change has not been noted in previous analyses, it is important to acknowledge that during severe water deficit, thousands of genes are non-specifically downregulated due to general cellular stress and metabolic slowdown [6], and without functional validation it is not possible to distinguish a specific regulatory response from a passive stress consequence. C2H2-ZFP promoters were enriched for ABRE elements (74.7% vs. genome-wide ~20%, *p* < 0.001) and DRE elements (47.9% vs. ~10%, *p* < 0.01; Figure 6D; Table S3). Biological replicate correlations were 0.85–0.97 (Table S8).

### 2.8. Marchantia C2H2-ZFPs Do Not Respond to Osmotic Stress

**Figure 7:**
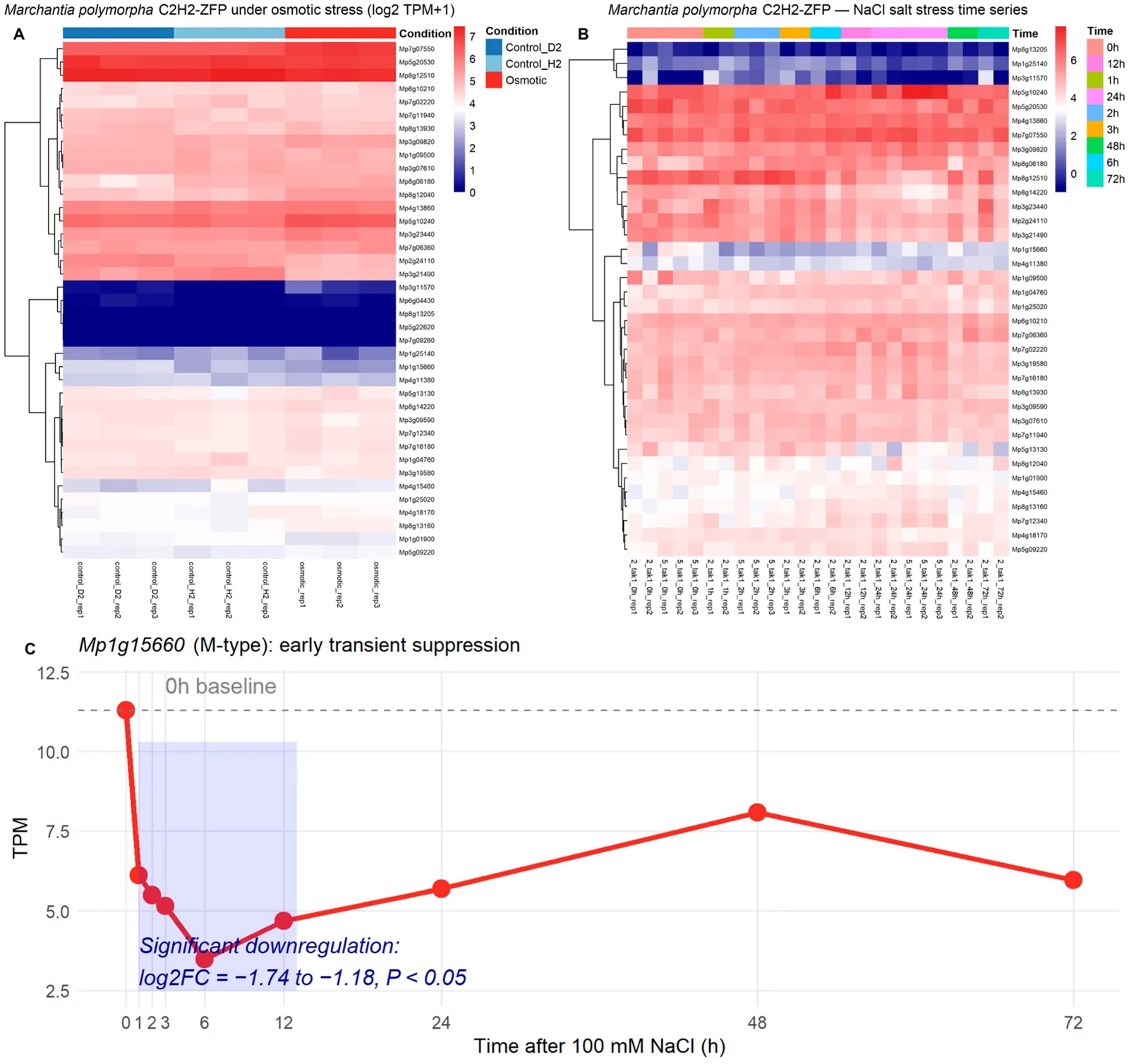
Marchantia polymorpha C2H2-ZFP expression under osmotic and salt stress. (**A**) Osmotic heatmap. (**B**) Salt heatmap. (**C**) Mp1g15660 time-course line plot.

Analysis of mannitol-treated *M. polymorpha* RNA-seq data [9] showed that of 39 C2H2-ZFP genes, 35 were expressed. No C2H2-ZFP gene was significantly differentially expressed in any comparison (Figure 7A; Table S9). Cross-referencing against the Tan et al. [9] catalog confirmed zero C2H2-ZFP genes among 731 mannitol-responsive genes. Of the 75 stress-responsive TFs identified, 9 belong to the C2H2 family, but none responded to mannitol.

### 2.9. Weak, Transient Salt Stress C2H2-ZFP Response in Marchantia

Salt stress (100 mM NaCl) time-series analysis [14] revealed a weak C2H2-ZFP transcriptional response (chosen to test whether the absence of an osmotic response extends to ionic stress, which engages a distinct signaling pathway) (Figure 7B,C; Table S10). Applying the strict DE criteria defined above, five unique C2H2-ZFP genes were responsive to salt at any sampled time point: four were downregulated and one was upregulated (Table S10). One gene—*Mp1g15660* (M-type, single zinc finger)—showed consistent downregulation at 2–12 h (log_2_ fold change = −1.74 to −1.18, *p* < 0.05), recovering by 24 h. At 24 h, *Mp8g12510* (log_2_ fold change = −1.72, *p* = 0.0002) and two other M-type genes (*Mp8g14220* and *Mp3g21490*) were significantly downregulated. The single upregulated gene was the M-type zinc finger *Mp8g06180* (3 h, log_2_ fold change = 1.00, *p* < 0.01). The sole Q-type gene showing any salt response, *Mp3g23440*, rose 2.0-fold at 1 h (log_2_ fold change = 1.02) but did not reach the significance threshold. No genes met late or sustained upregulation criteria.

### 2.10. Four-Condition Comparison

**Figure 8:**
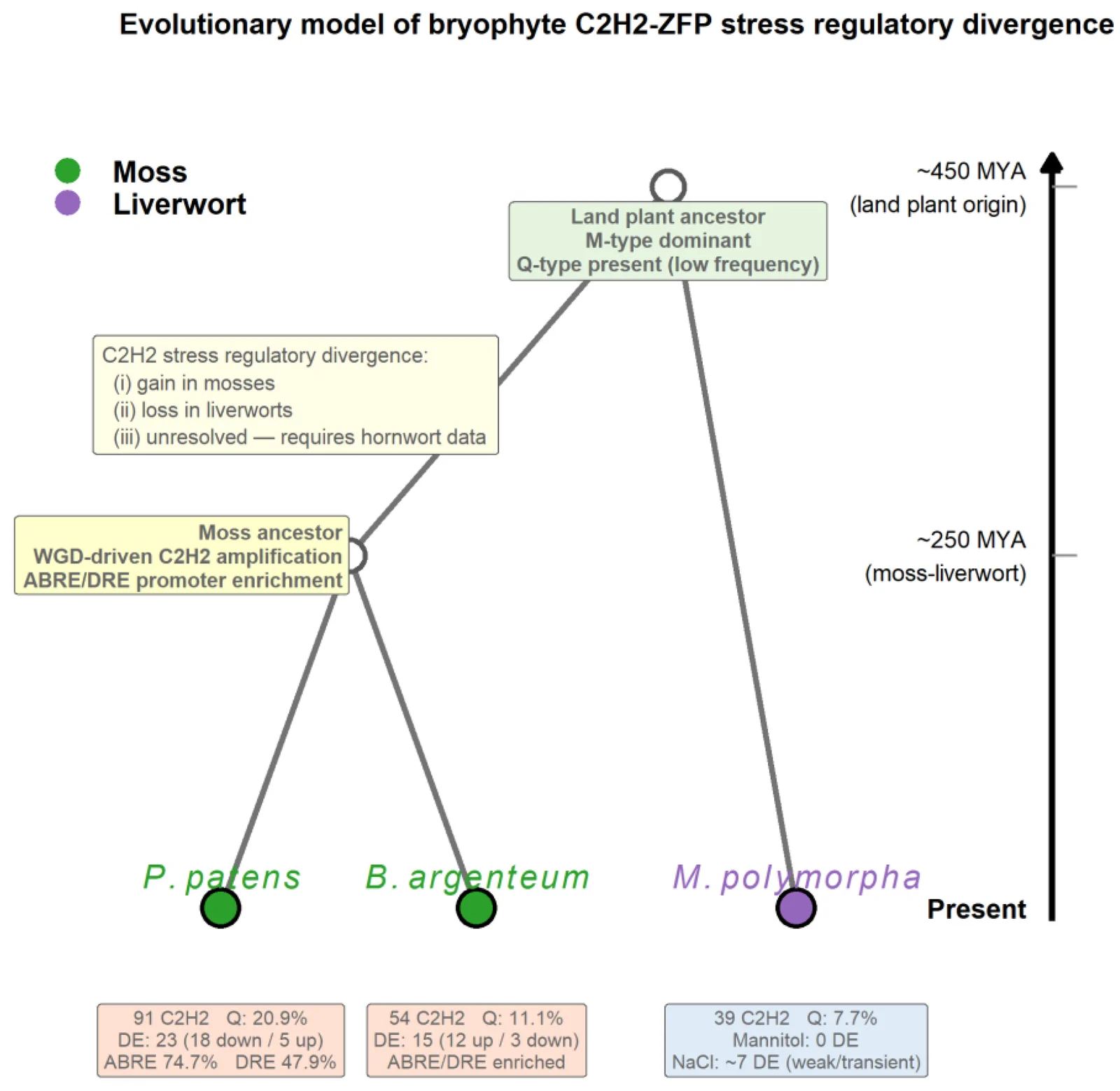
Evolutionary model of bryophyte C2H2-ZFP stress regulatory divergence. Vertical schematic showing the three species at tips, with inferred evolutionary events at internal nodes.

Integration of all four datasets revealed a phylogenetic pattern (Figure 8; Table 1 and Table S11). Mosses mobilized 25–28% of their C2H2-ZFP repertoire under dehydration. *Marchantia* showed no significant C2H2-ZFP differential expression under pure osmotic stress and only a marginal, transient response to combined osmotic + ionic stress (NaCl). This lineage-specific dichotomy indicates that C2H2-ZFP-mediated osmotic and salt stress transcriptional regulation has diverged substantially between moss and liverwort lineages.

## 3. Discussion

The pan-bryophyte TF survey suggests that mosses carry a regulatory toolkit comparable in complexity to that of vascular plants. Thirty-five TF families were significantly expanded in mosses relative to liverworts. The B3 domain family showed the strongest moss-specific expansion, consistent with evolutionary investment in dehydration-responsive regulatory machinery [16,17].

Domain-level C2H2-ZFP classification yields quantitatively different results from protein-level classification. The 67.8% BLASTP-HMMER recall for C2H2 reflects that this is among the most structurally diverse TF families and breadth-layer BLASTP (E ≤ 1 × 10^−10^) is conservative for divergent zinc-finger paralogs; depth-layer HMMER re-classification supersedes breadth counts for the C2H2 family.

The most informative functional result appears to be the absence of significant C2H2-ZFP osmotic stress responsiveness in *M. polymorpha* under stringent criteria, contrasting with strong C2H2-ZFP mobilization in both mosses (23 genes in *P. patens*, 15 genes in *B. argenteum*). These results are consistent with differential evolution of C2H2-ZFP-mediated osmotic and dehydration stress transcriptional regulation between mosses and liverworts. Two scenarios, not mutually exclusive, could explain this pattern: (i) gain in mosses, or (ii) loss in liverworts. Discriminating between these hypotheses requires stress expression data from hornworts and charophyte algae.

Re-analysis of the published *P. patens* dehydration RNA-seq dataset [13] showed that *STOP1* (Pp26Ghq00168) transcript levels were reduced (log_2_ fold change = −1.42) under severe dehydration. *PpSTOP2* (Pp13Ghq00841), a C2H2-ZFP homolog of *PpSTOP1* recently shown to activate the cation channel gene *PpCSC1* under salt stress [18], was also detectable in the same dehydration dataset and declined modestly (log_2_ fold change = −0.86), falling below our differential expression threshold of |log_2_ fold change| > 1. This observation should be interpreted cautiously: during severe water deficit, broad transcriptional reprogramming occurs, and the downregulation of individual genes may reflect general metabolic suppression rather than a specific, adaptive regulatory response [6]. Whether the altered *STOP1* and *PpSTOP2* expression levels contribute causally to dehydration physiology—for example, through modulation of aluminum-tolerance pathways or cross-talk with other stress signaling cascades—remains to be determined. Functional studies, such as the generation of knockout and overexpression lines in *P. patens* followed by dehydration phenotyping and transcriptomic profiling, would be required to establish whether these expression changes represent dedicated regulatory mechanisms or non-specific consequences of cellular stress. Moreover, since *STOP1* functions as a positive regulator, its downregulation would be expected to lead to a passive decline of its downstream targets, including *PpSTOP2*, further supporting the interpretation that these expression changes may reflect a consequence of general stress rather than a specific regulatory event.

The ~5-fold entropy difference between the QALGGH core hexapeptide (0.34 bits) and its flanking residues (1.68 bits) suggests that this motif ranks among the most strongly conserved plant TF DNA-binding motifs known [19].

### 3.1. C2H2-ZFP Stress Responsiveness in Land Plant Evolution

The contrasting C2H2-ZFP stress expression patterns observed between mosses and *M. polymorpha* raise broader questions about the evolution of stress-regulatory networks across land plants. In angiosperms, C2H2-ZFPs are well-established regulators of abiotic stress tolerance: *AtZAT6* and *AtZAT10* modulate salt and osmotic stress responses in *Arabidopsis* [11], while rice *ZFP182* and *ZFP36* enhance drought tolerance when overexpressed [12]. The observation that 25–28% of moss C2H2-ZFPs are mobilized under dehydration—a proportion comparable to that seen in angiosperm drought transcriptomes—suggests that mosses may have independently evolved or ancestrally retained a robust C2H2-ZFP-mediated dehydration regulatory network. Conversely, the near-complete absence of C2H2-ZFP osmotic responsiveness in *M. polymorpha* is unusual and warrants further investigation. One possibility is that liverworts rely more heavily on other TF families for osmotic stress signaling; Tan et al. [9] reported that bZIP, MYB, and NAC family TFs were prominently represented among mannitol-responsive genes in *M. polymorpha*, suggesting that the regulatory architecture of osmotic stress responses may differ fundamentally between the two bryophyte lineages.

The desiccation tolerance of *B. argenteum* provides an additional dimension for interpreting these results. Unlike *P. patens*, which is desiccation-sensitive at the vegetative stage, *B. argenteum* can survive near-complete dehydration [20]. The upregulation of 12 of 15 dehydration-responsive C2H2-ZFPs in *B. argenteum* (versus only 5 of 23 in *P. patens*) suggests that positive regulation of C2H2-ZFPs may contribute to desiccation tolerance, potentially through activation of protective downstream pathways. The functional characterization of *BaZFP1*, which promotes growth in heterologous assays [15], supports this hypothesis. Comparative functional genomics between desiccation-tolerant and desiccation-sensitive mosses could help identify the specific C2H2-ZFP members and downstream targets that distinguish these two adaptive strategies.

The expansion of C2H2-ZFP gene families in mosses, driven partly by WGD, may have provided the raw genetic material for the evolution of lineage-specific stress regulatory networks. This pattern parallels observations in angiosperms, where polyploidy events have been associated with the expansion and functional diversification of stress-related TF families, including C2H2-ZFPs [2,3]. The retention of duplicated C2H2-ZFP copies in moss genomes, combined with the enrichment of stress-responsive cis-elements (ABRE, DRE) in C2H2-ZFP promoters, is consistent with subfunctionalization following WGD. Future work integrating chromatin accessibility data (ATAC-seq) and genome-wide TF binding profiles (DAP-seq) across bryophyte species would help resolve whether the observed expression divergence reflects changes in cis-regulatory architecture or in the upstream signaling pathways that converge on C2H2-ZFP promoters.

### 3.2. Limitations and Future Directions

This study is a hypothesis-generating comparative re-analysis of public genomic and transcriptomic resources. Functional validation of the 23 dehydration-responsive C2H2-ZFP candidates in *P. patens*, particularly *STOP1*, through CRISPR-Cas9 mutagenesis would be required to establish causality. The promoter cis-element analysis was restricted to *P. patens*; a systematic comparison of C2H2-ZFP promoter architecture across all three species would strengthen the cis-regulatory argument. The differential moss–liverwort C2H2 stress response hypothesis should be tested in hornworts. While functional validation was beyond the scope of this comparative re-analysis, the 23 dehydration-responsive *P. patens* C2H2-ZFP genes represent high-priority candidates for follow-up studies.

## 4. Materials and Methods

### 4.1. Genome Data Acquisition

Protein-coding gene annotations for 123 bryophyte genomes [7] (4 hornworts, 37 liverworts, 82 mosses) were obtained from bryogenomes.org. An additional 21 published genomes were downloaded, including *P. patens* T2T [21], *M. polymorpha* v7.1 [22], and *B. argenteum* [23] (NCBI Assembly GCA_037043015.1). The combined dataset comprised 4,040,893 protein sequences.

### 4.2. Breadth Layer: TF Family Classification

PlantTFDB v5.0 reference TF protein sets were used to construct a combined reference of 6226 curated plant TFs. All proteins from the combined 144-genome dataset were queried using BLASTP 2.16.0 (E-value ≤ 1 × 10^−10^, top hit). Welch’s *t*-test with Benjamini–Hochberg correction was applied (*p* < 0.01, |log_2_ fold change| > 0.5).

### 4.3. Depth Layer: C2H2-ZFP Annotation and Classification

HMMER 3.4 with Pfam 37.0 models PF00096 and PF13912 (E-value ≤ 1 × 10^−3^) was used for C2H2 domain identification. Domains were cross-validated with InterProScan 5.59-91.0, SMART, and NCBI-CDD. Classification into M-, Z-, Q-, and D-types followed Li et al. [13] criteria. For independent validation, a full-protein C2H2 motif scan was applied to all 8246 C2H2-ZFP genes, yielding 11,565 zinc fingers. Cross-method agreement was 90.7%.

### 4.4. Phylogenetic and Evolutionary Analyses

Protein sequences were clustered at 70% identity (CD-HIT 4.8.1; 2869 representatives), then at 50% identity (1518 representatives), subsampled to 467 sequences, aligned with MAFFT v7.520, trimmed with trimAl v1.5.rev1, and analyzed with IQ-TREE 2 v2.3.6 under VT+F+R8. MEME Suite v5.5.5 identified conserved motifs. OrthoFinder v3.1.5 [24] identified 53,068 orthogroups. QALGGH motif conservation used position-specific entropy analysis of 1153 Q-type motifs. Genome size–C2H2 count correlation used Pearson correlation (*n* = 14). Duplication modes were assigned to paralogous pairs following the criteria of Wang et al. [25]: tandem duplicates were defined as paralogs separated by five or fewer intervening genes on the same chromosome; segmental duplicates were those located in syntenic blocks identified by MCScanX v1.0.0 (default parameters, E-value ≤ 1 × 10^−10^); all remaining paralogs were classified as dispersed. For cis-regulatory element enrichment, 1500 bp promoter sequences upstream of the transcription start site were extracted and scanned with FIMO (MEME Suite v5.5.5) against the PLACE database; motif enrichment relative to the genome-wide background was tested with a hypergeometric test followed by Benjamini–Hochberg correction.

### 4.5. Transcriptome Integration

All stress treatments whose transcriptomic data we analyzed were performed in the original studies cited: severe dehydration (desiccation) of *P. patens* [13] and *B. argenteum* [20]; osmotic (mannitol) stress of *M. polymorpha* [9]; and salt (100 mM NaCl) time-series treatment of *M. polymorpha* [14]. Dehydration was assayed in the two moss species because published dehydration RNA-seq datasets were available for them, whereas the publicly available *M. polymorpha* stress atlas [9] applies mannitol as its osmotic-stress treatment; mannitol was therefore used as the closest available osmotic analog for the liverwort. RNA-seq data were aligned with HISAT2 2.1.0, quantified with featureCounts v2.0.8, and TPM-normalized. Differential expression used Welch’s *t*-test (|log_2_ fold change| > 1, *p* < 0.05). Each dataset was processed separately through an identical pipeline and analyzed against its own controls; no cross-dataset batch correction was applied, and DE was defined per dataset with the same criteria above.

## 5. Conclusions

This study presents a comprehensive transcription factor atlas across 144 bryophyte genomes, with deep evolutionary characterization of the C2H2 zinc-finger protein family. Our analysis suggests that domain-level classification substantially revises Z-type frequency from ~20% to 4.2%, that Q-type radiation occurred primarily in seed plants, and that C2H2-ZFP-mediated stress responses appear to have diverged markedly between moss and liverwort lineages. The dehydration-responsive expression of *STOP1* may extend its known functional contexts beyond aluminum tolerance, although functional validation is needed to confirm this. The nested analytical framework established here—breadth classification followed by depth dissection of an exemplar family—provides a template for studying other bryophyte-expanded TF families. All classification tables, expression matrices, and phylogenetic outputs are provided as a community resource.

## Figures and Tables

**Table 1:** C2H2-ZFP stress responsiveness across bryophyte species and stress conditions.

Species	Lineage	Stress	C2H2 Genes	DE Genes	Direction	Response
*P. patens*	Moss	Dehydration	91	23 (25.3%)	18 ↓/5 ↑	Strong
*B. argenteum*	Moss	Dehydration	54	15 (27.8%)	3 ↓/12 ↑	Strong
*M. polymorpha*	Liverwort	Mannitol	39	0 (0%)	—	None
*M. polymorpha*	Liverwort	NaCl	39	5 (12.8%)	4 ↓/1 ↑	Weak/transient

*Arrows indicate the number of downregulated* (↓) *and upregulated* (↑) *differentially expressed C2H2-ZFP genes under each stress condition*.

## Data Availability

The publicly available datasets re-analyzed in this study can be found at: Bryophyte genomes (123 species: 82 mosses, 37 liverworts, 4 hornworts): BryoGenomes consortium (https://bryogenomes.org); Outgroup land plant genomes: Phytozome v13 (https://phytozome.jgi.doe.gov, accessed on 5 August 2026) and NCBI RefSeq; *Physcomitrium patens* dehydration RNA-seq: NCBI BioProject PRJNA800025; *Bryum argenteum* dehydration RNA-seq: NCBI BioProject PRJNA327617; *Marchantia polymorpha* osmotic stress RNA-seq: EBI ArrayExpress E-MTAB-11141; *Marchantia polymorpha* salt stress RNA-seq: NCBI BioProject PRJNA641427.

## Supplementary Materials

The following supporting information can be downloaded at: https://www.mdpi.com/article/10.3390/plants15162452/s1.

## Abbreviations

The following abbreviations are used in this manuscript:

ABRE	ABA-responsive element
C2H2-ZFP	C2H2 zinc-finger protein
DE	differentially expressed
DRE	dehydration-responsive element
HMMER	profile hidden Markov model-based sequence analysis software
TF	transcription factor
TPM	transcripts per million
WGD	whole-genome duplication
