# Supplementary material for: A Bryophyte Transcription Factor Landscape Reveals Lineage-Specific Zinc-Finger Protein Evolution and Differential Stress Mobilization Between Mosses and Liverworts
Source: Plants (Basel). 2026 Aug 12;15(16):2452. doi: 10.3390/plants15162452 (PMC13516842; doi:10.3390/plants15162452)
Supplement: Supplementary file 1 [file plants-15-02452-s001.zip › plants-4468665-supplementary.pdf]

Article

A Bryophyte Transcription Factor Landscape Reveals Lineage-Specific Zinc-Finger Protein Evolution and Differential Stress Mobilization Between Mosses and Liverworts

Supplementary Figures

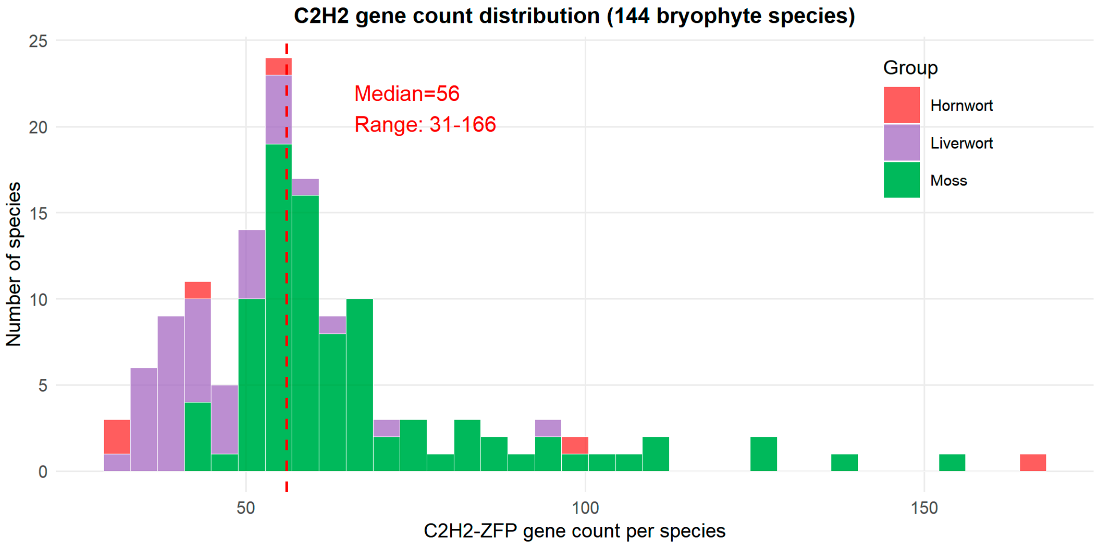

Figure S1. Species distribution of C2H2-ZFP gene counts across 144 bryophyte genomes.

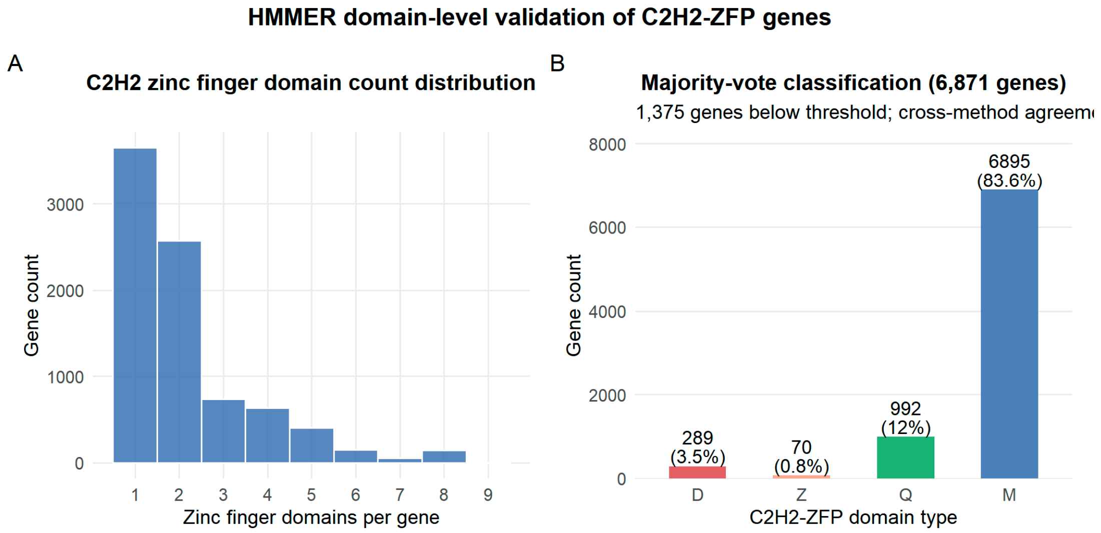

Figure S2. HMMER domain-level validation of C2H2-ZFP genes. (A) Distribution of zinc finger domain counts per gene. (B) Majority-vote classification results.

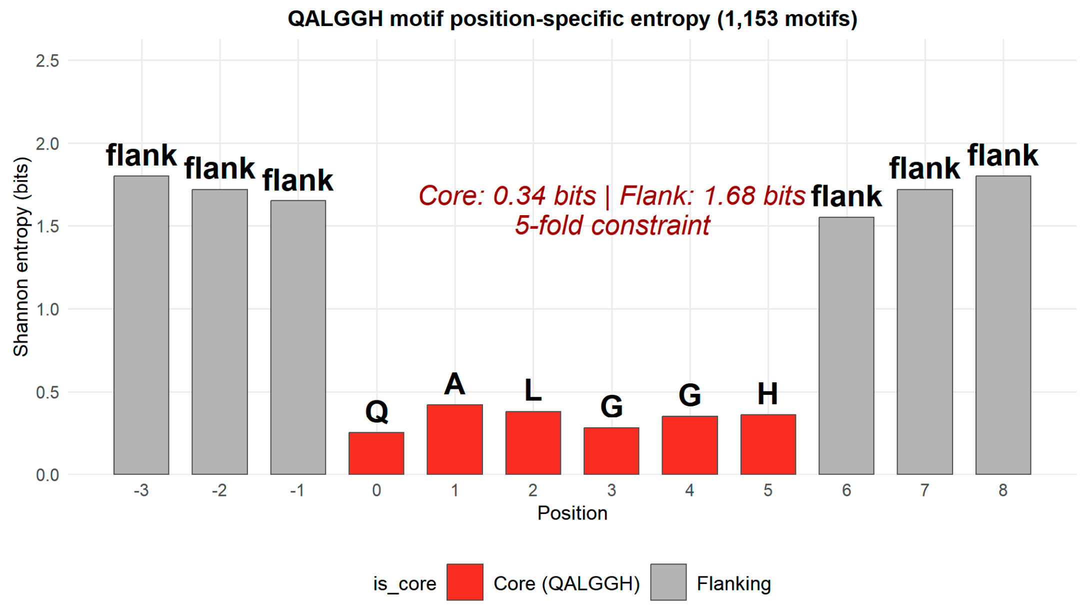

Figure S3. Position-specific entropy of the QALGGH motif across 1,153 Q-type zinc finger domains.

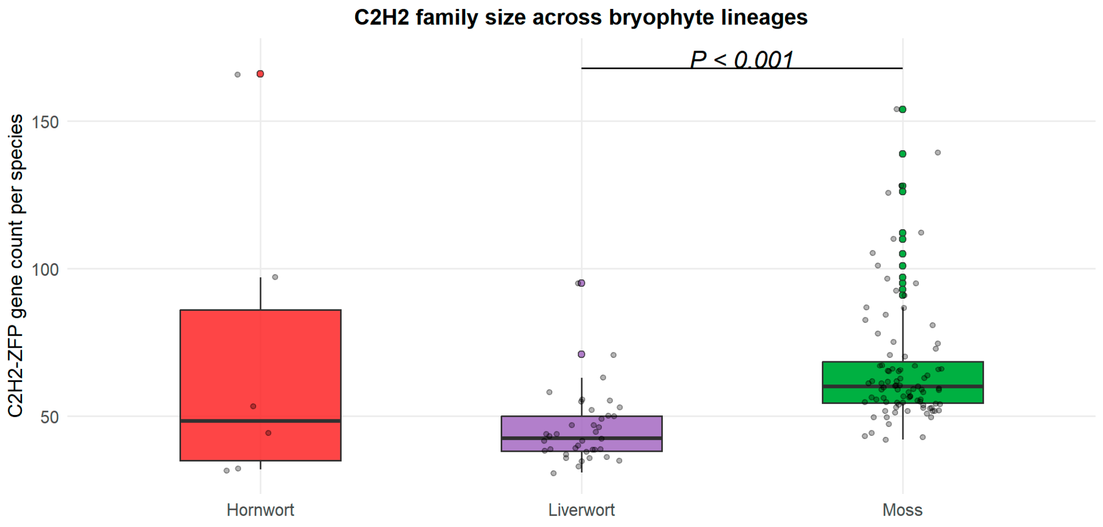

Figure S4. C2H2-ZFP family size across bryophyte lineages (moss, liverwort, hornwort).

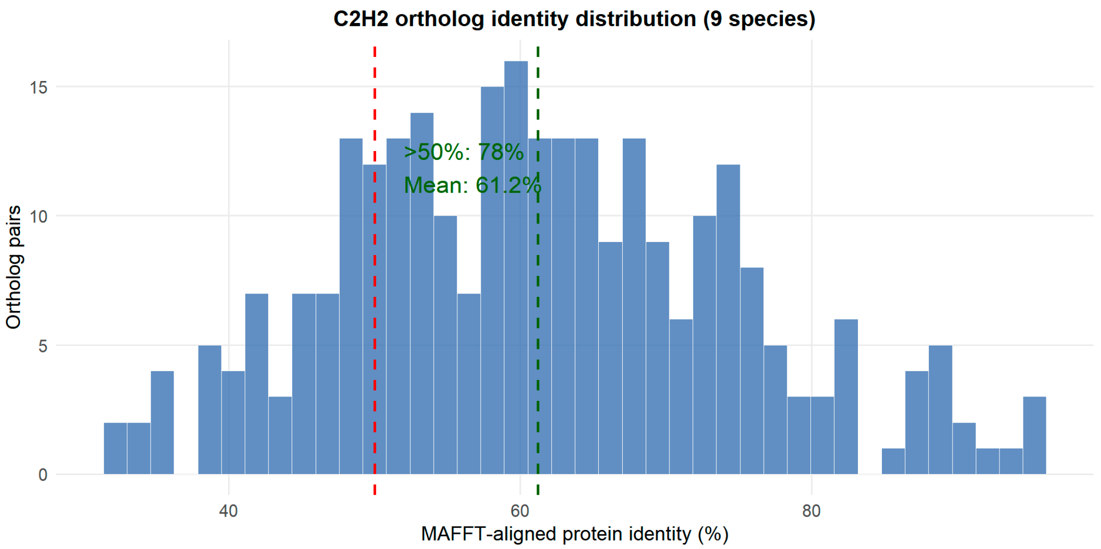

Figure S5. Distribution of protein sequence identity among C2H2-ZFP ortholog pairs across nine representative bryophyte species.

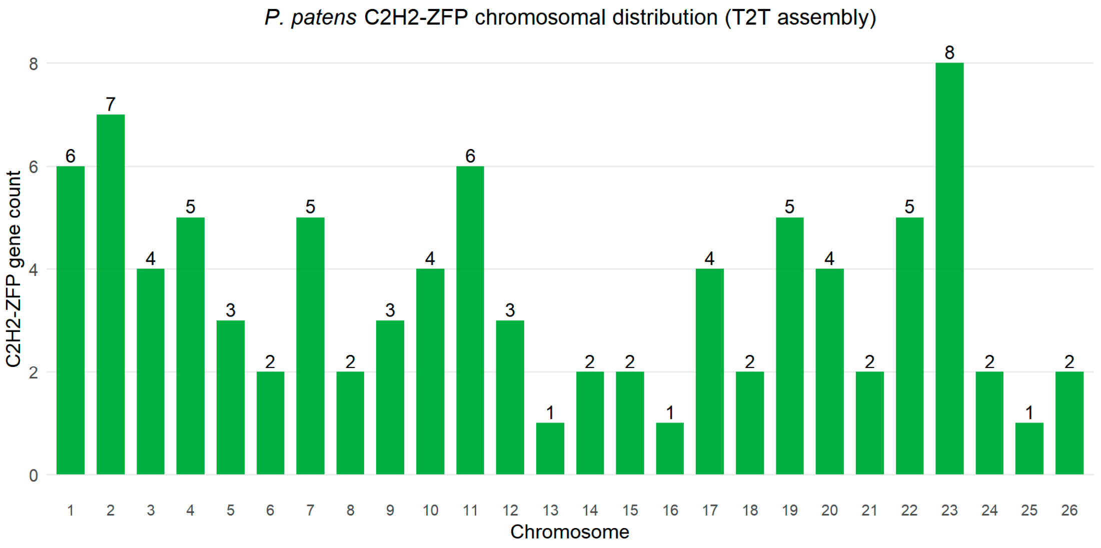

Figure S6. Chromosomal distribution of C2H2-ZFP genes in the *Physcomitrium patens* T2T genome assembly.

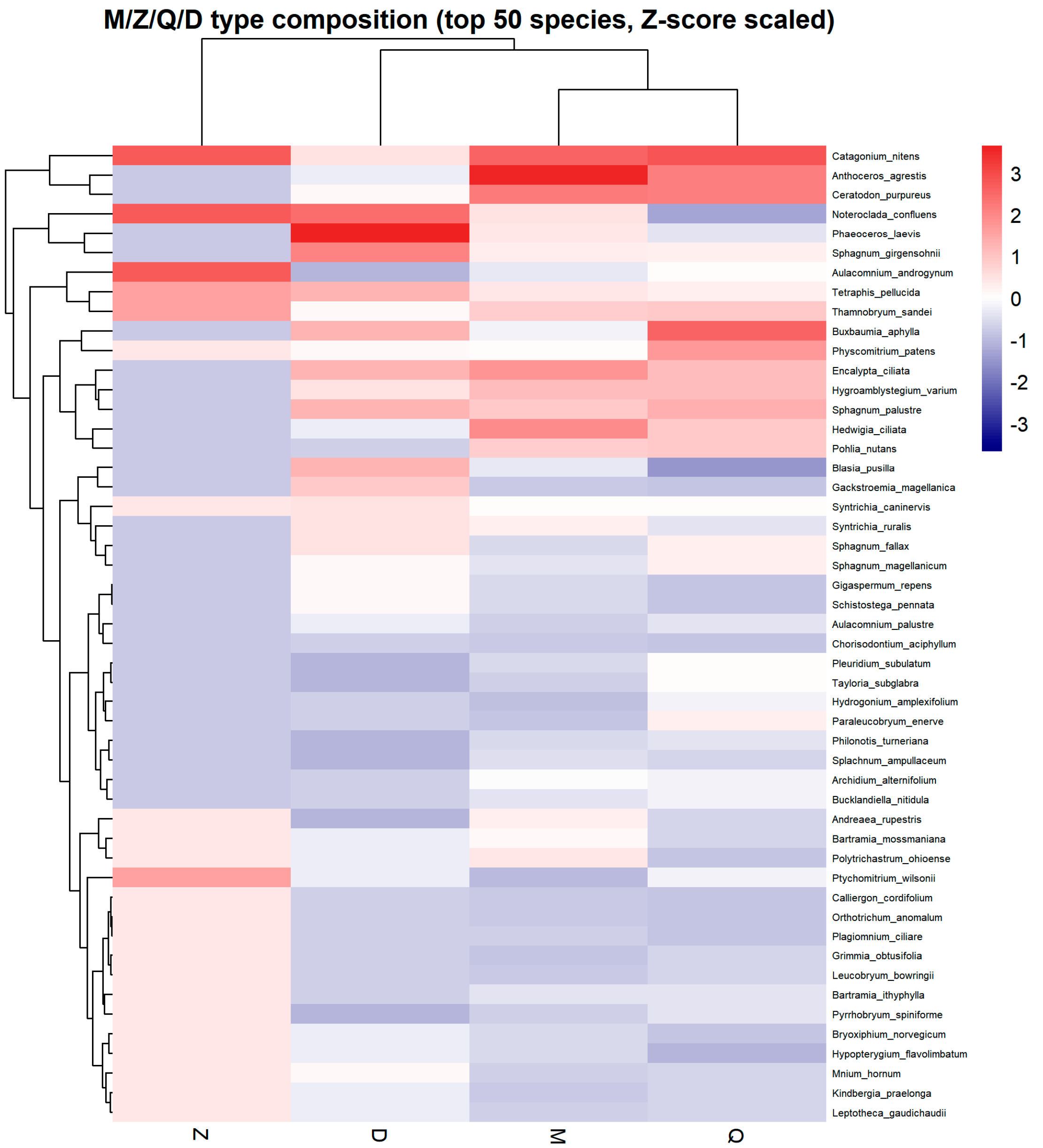

Figure S7. M/Z/Q/D-type composition heatmap across the top 50 bryophyte genomes ranked by C2H2-ZFP gene count.
